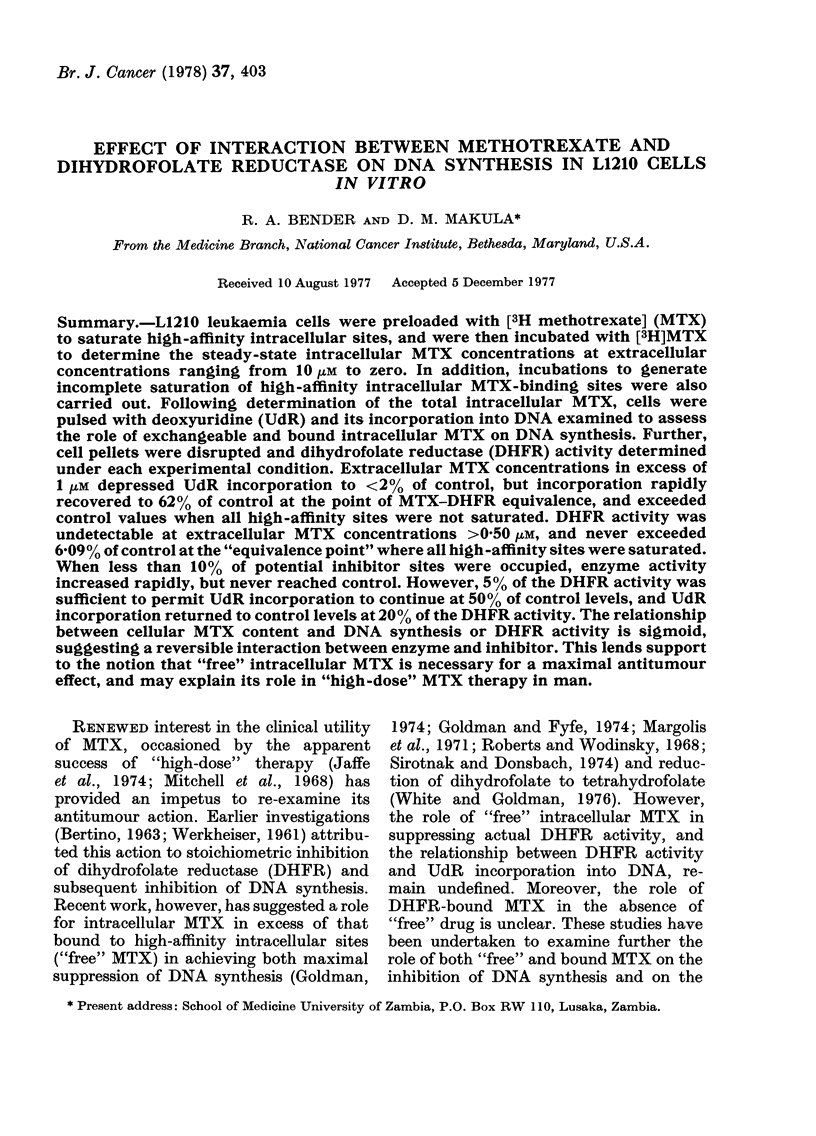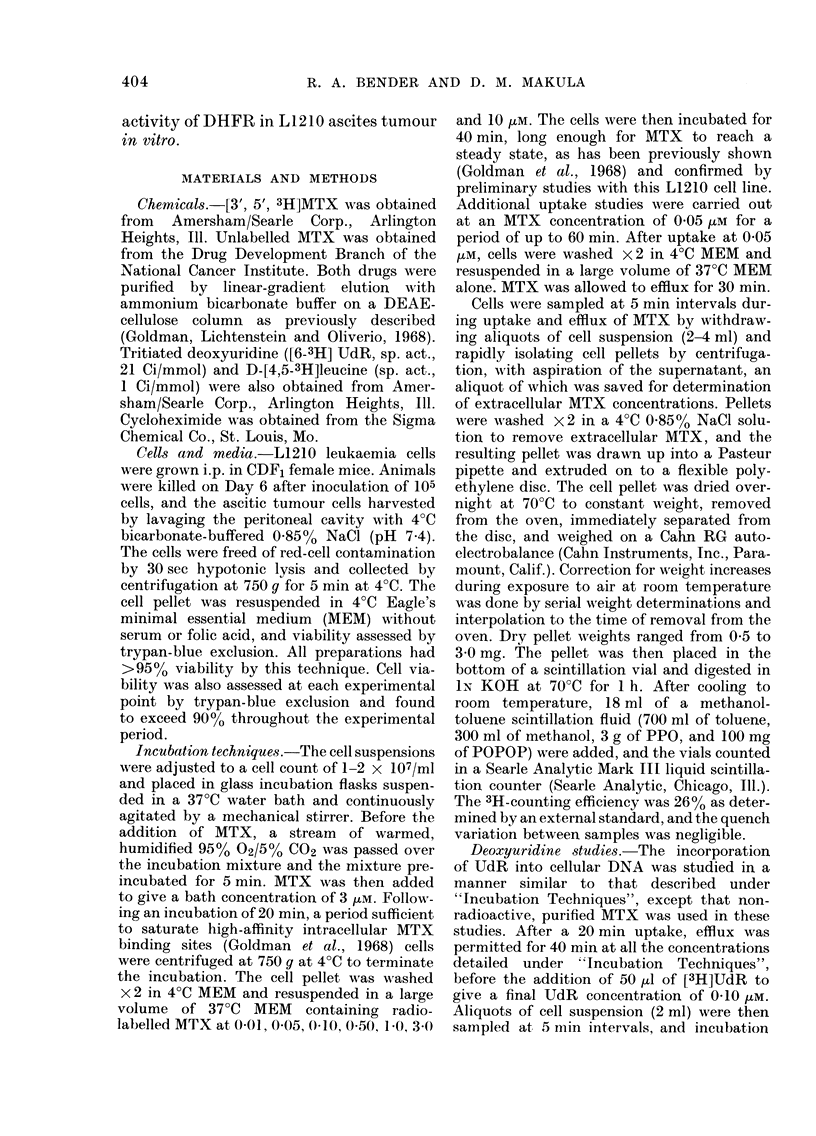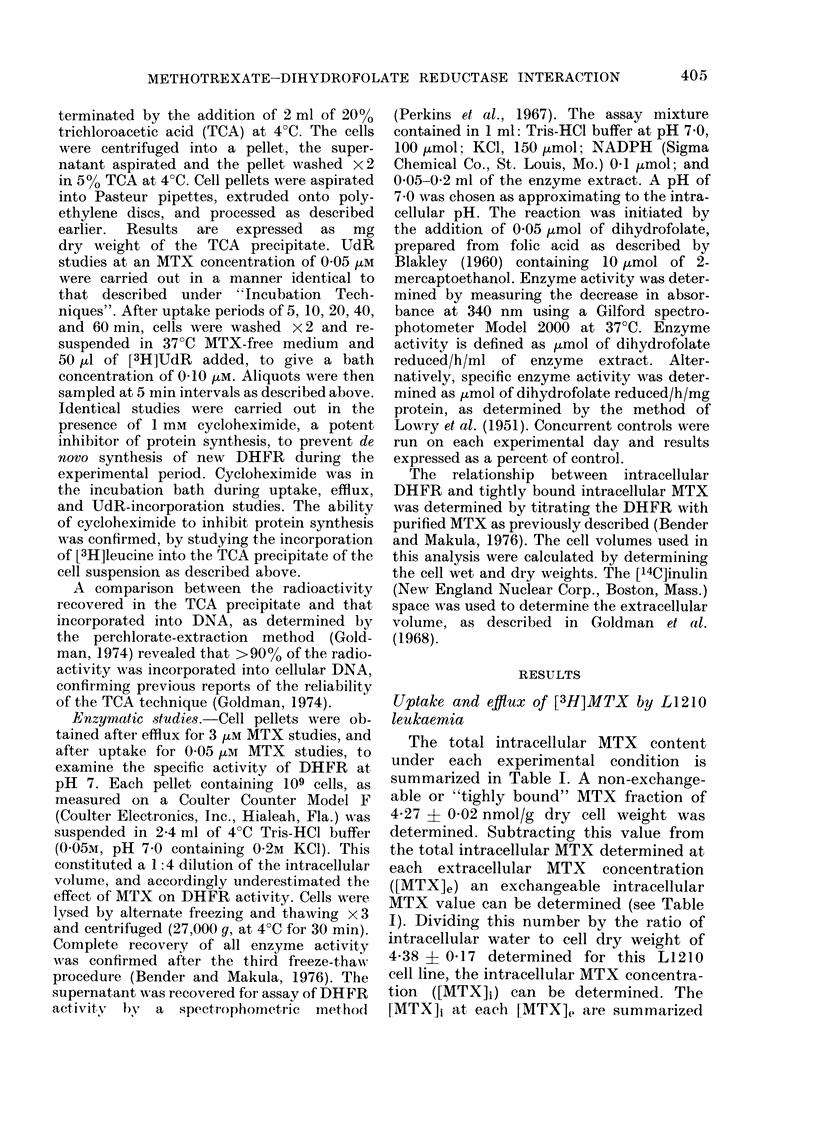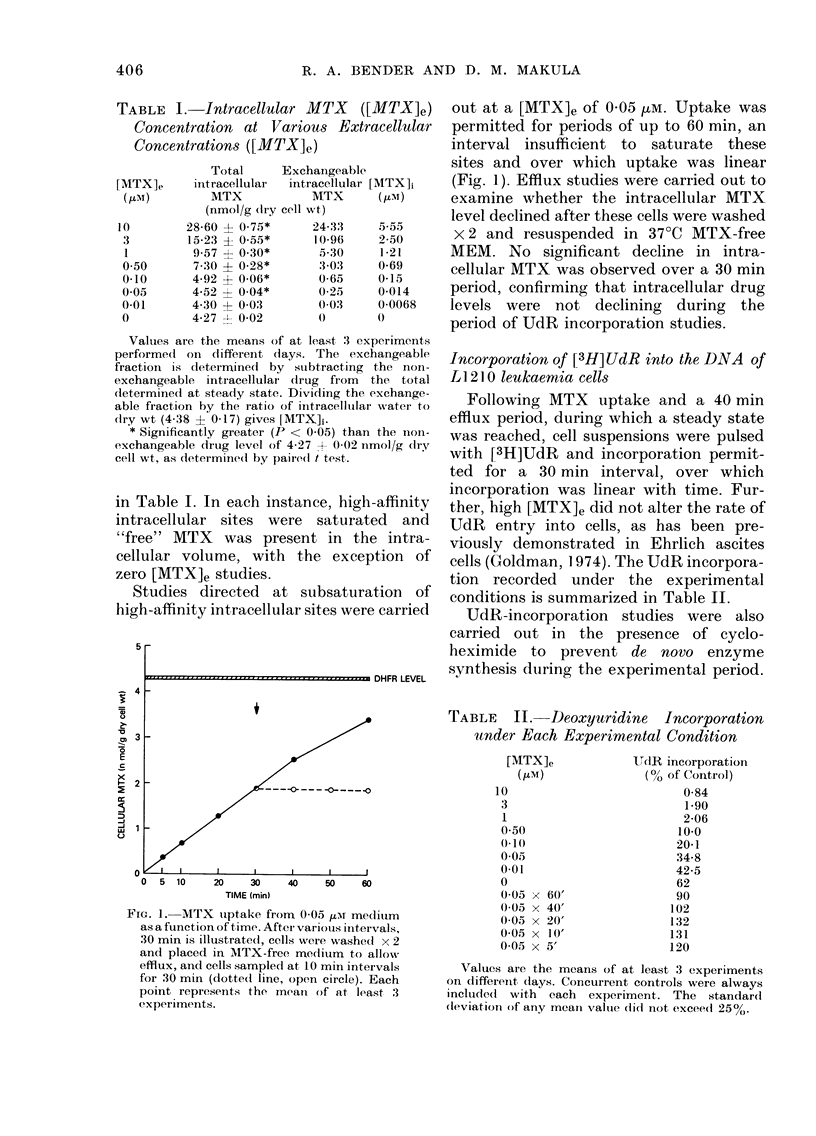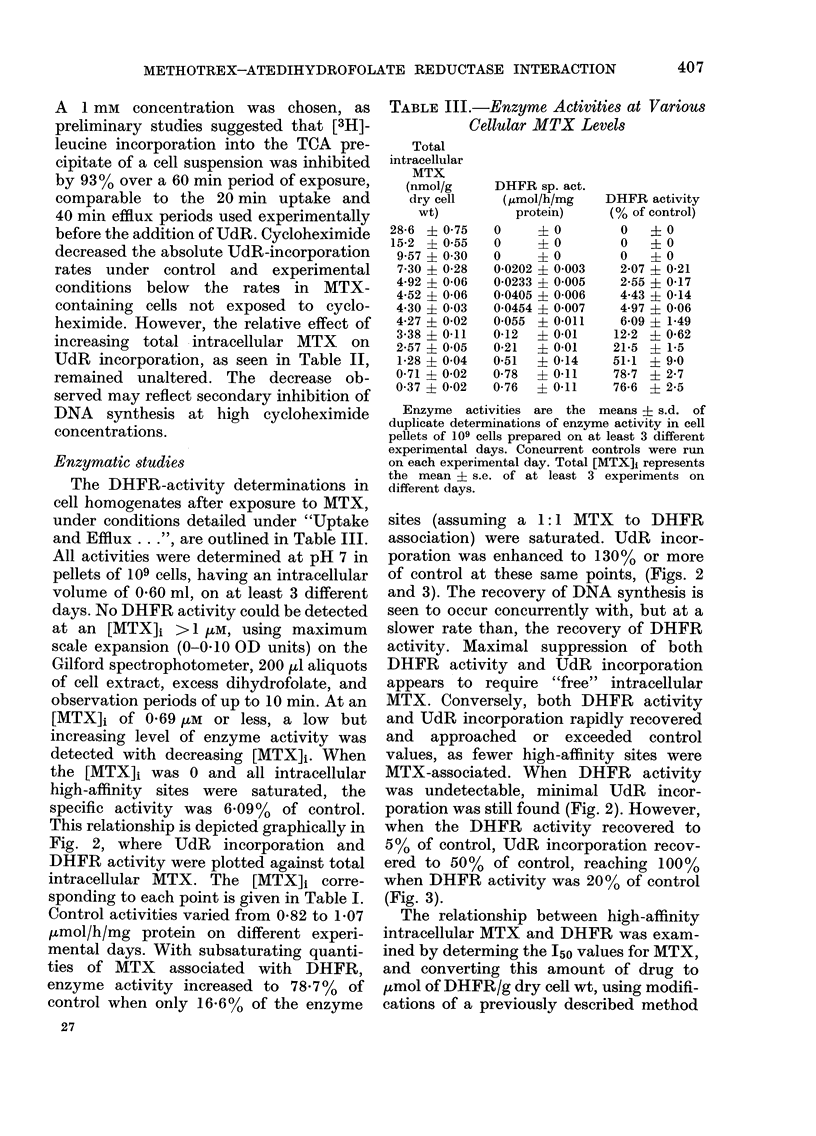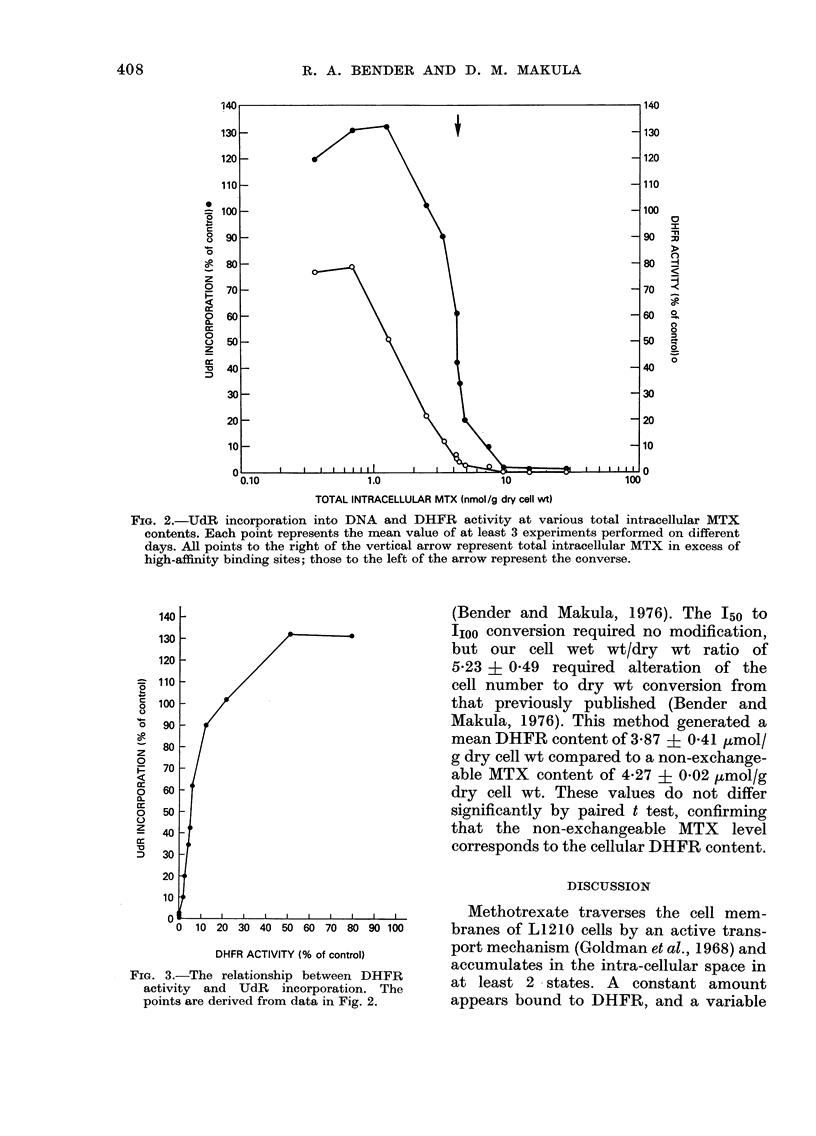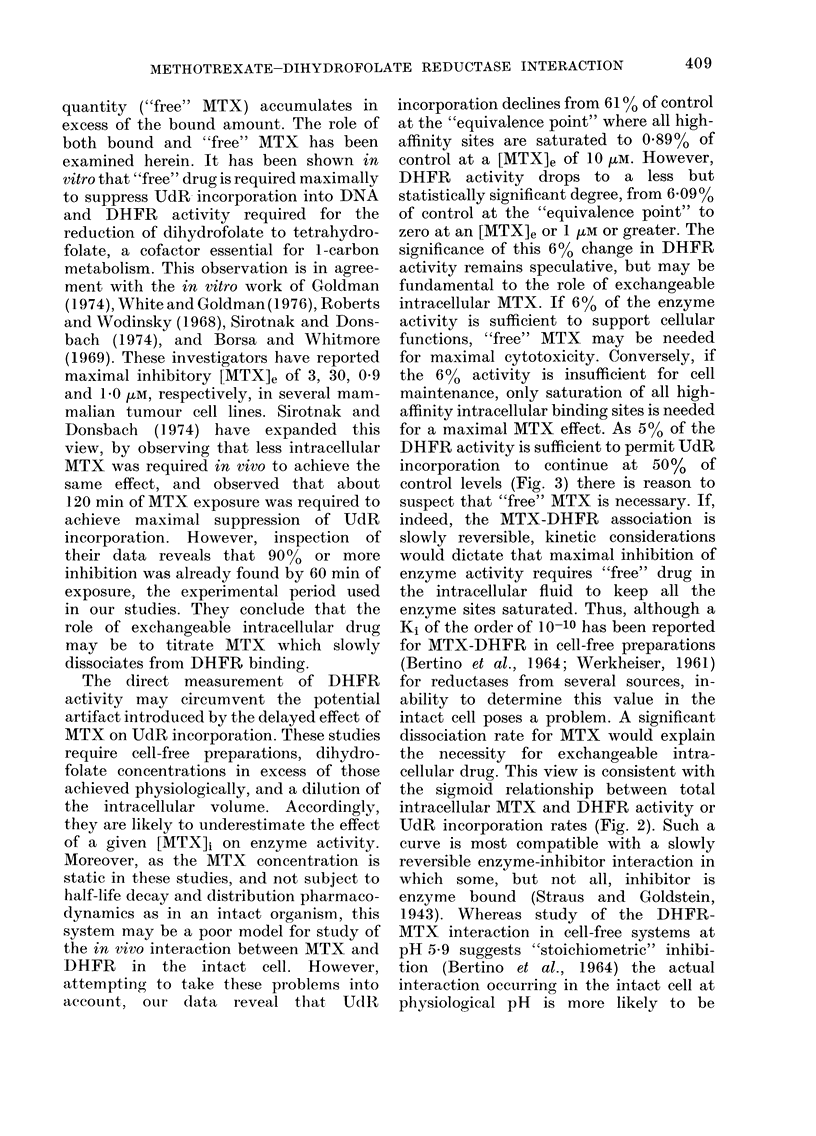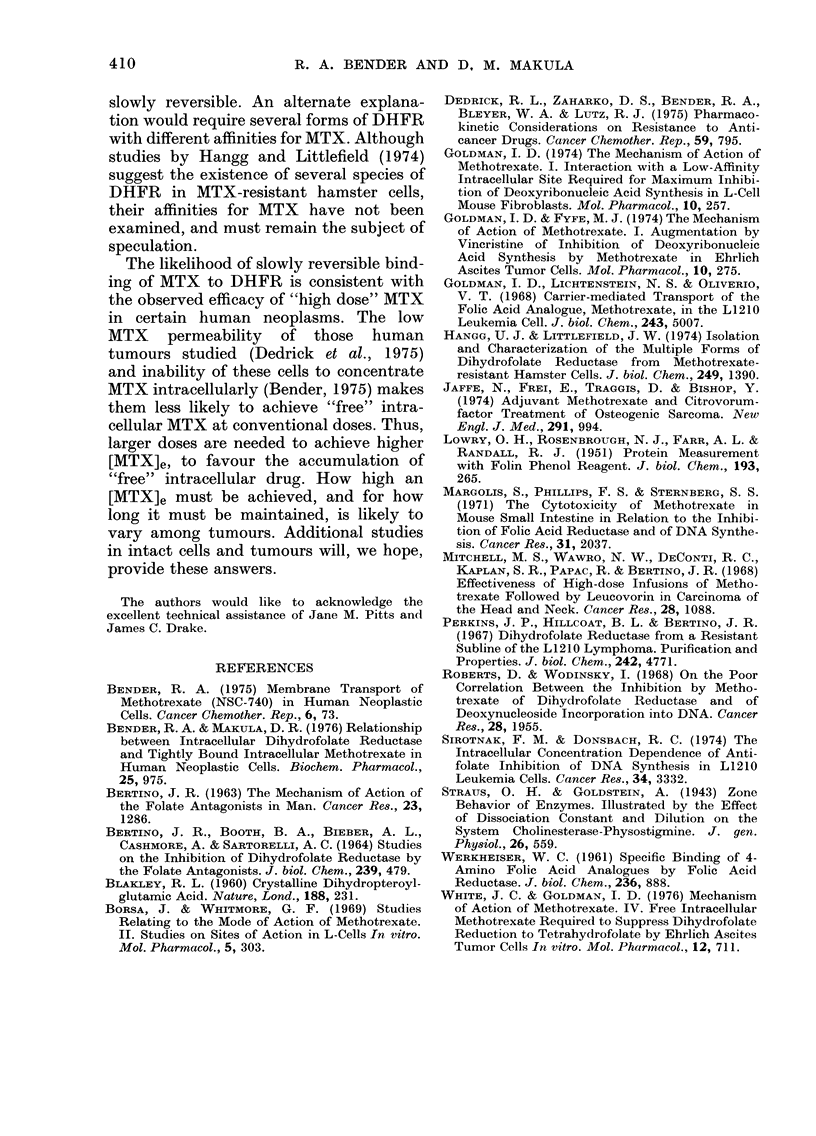# Effect of Interaction Between Methotrexate and Dihydrofolate Reductase on DNA Synthesis in L1210 Cells In Vitro

**DOI:** 10.1038/bjc.1978.60

**Published:** 1978-03

**Authors:** R. A. Bender, D. M. Makula

## Abstract

L1210 leukaemia cells were preloaded with [^3^H methotrexate] (MTX) to saturate high-affinity intracellular sites, and were then incubated with [^3^H]MTX to determine the steady-state intracellular MTX concentrations at extracellular concentrations ranging from 10 μM to zero. In addition, incubations to generate incomplete saturation of high-affinity intracellular MTX-binding sites were also carried out. Following determination of the total intracellular MTX, cells were pulsed with deoxyuridine (UdR) and its incorporation into DNA examined to assess the role of exchangeable and bound intracellular MTX on DNA synthesis. Further, cell pellets were disrupted and dihydrofolate reductase (DHFR) activity determined under each experimental condition. Extracellular MTX concentrations in excess of 1 μM depressed UdR incorporation to <2% of control, but incorporation rapidly recovered to 62% of control at the point of MTX-DHFR equivalence, and exceeded control values when all high-affinity sites were not saturated. DHFR activity was undetectable at extracellular MTX concentrations >0.50 μM, and never exceeded 6.09% of control at the “equivalence point” where all high-affinity sites were saturated. When less than 10% of potential inhibitor sites were occupied, enzyme activity increased rapidly, but never reached control. However, 5% of the DHFR activity was sufficient to permit UdR incorporation to continue at 50% of control levels, and UdR incorporation returned to control levels at 20% of the DHFR activity. The relationship between cellular MTX content and DNA synthesis or DHFR activity is sigmoid, suggesting a reversible interaction between enzyme and inhibitor. This lends support to the notion that “free” intracellular MTX is necessary for a maximal antitumour effect, and may explain its role in “high-dose” MTX therapy in man.


Br. J. Cancer (1978) 37, 403

### EFFECT OF INTERACTION BETWEEN METHOTREXATE AND DIHYDROFOLATE REDUCTASE ON DNA SYNTHESIS IN L1210 CELLS *IN VITRO*

R. A. BENDER AND D. M. MAKULA*

*From the Medicine Branch, National Cancer Institute, Bethesda, Maryland, U.S.A.*

Received 10 August 1977 Accepted 5 December 1977

**Summary.—L1210 leukaemia cells were preloaded with [$^3$H methotrexate] (MTX) to saturate high-affinity intracellular sites, and were then incubated with [$^3$H]MTX to determine the steady-state intracellular MTX concentrations at extracellular concentrations ranging from 10 $\mu$M to zero. In addition, incubations to generate incomplete saturation of high-affinity intracellular MTX-binding sites were also carried out. Following determination of the total intracellular MTX, cells were pulsed with deoxyuridine (UdR) and its incorporation into DNA examined to assess the role of exchangeable and bound intracellular MTX on DNA synthesis. Further, cell pellets were disrupted and dihydrofolate reductase (DHFR) activity determined under each experimental condition. Extracellular MTX concentrations in excess of 1 $\mu$M depressed UdR incorporation to <2% of control, but incorporation rapidly recovered to 62% of control at the point of MTX–DHFR equivalence, and exceeded control values when all high-affinity sites were not saturated. DHFR activity was undetectable at extracellular MTX concentrations >0·50 $\mu$M, and never exceeded 6·09% of control at the "equivalence point" where all high-affinity sites were saturated. When less than 10% of potential inhibitor sites were occupied, enzyme activity increased rapidly, but never reached control. However, 5% of the DHFR activity was sufficient to permit UdR incorporation to continue at 50% of control levels, and UdR incorporation returned to control levels at 20% of the DHFR activity. The relationship between cellular MTX content and DNA synthesis or DHFR activity is sigmoid, suggesting a reversible interaction between enzyme and inhibitor. This lends support to the notion that "free" intracellular MTX is necessary for a maximal antitumour effect, and may explain its role in "high-dose" MTX therapy in man.**

RENEWED interest in the clinical utility of MTX, occasioned by the apparent success of "high-dose" therapy (Jaffe *et al.*, 1974; Mitchell *et al.*, 1968) has provided an impetus to re-examine its antitumour action. Earlier investigations (Bertino, 1963; Werkheiser, 1961) attributed this action to stoichiometric inhibition of dihydrofolate reductase (DHFR) and subsequent inhibition of DNA synthesis. Recent work, however, has suggested a role for intracellular MTX in excess of that bound to high-affinity intracellular sites ("free" MTX) in achieving both maximal suppression of DNA synthesis (Goldman, 1974; Goldman and Fyfe, 1974; Margolis *et al.*, 1971; Roberts and Wodinsky, 1968; Sirotnak and Donsbach, 1974) and reduction of dihydrofolate to tetrahydrofolate (White and Goldman, 1976). However, the role of "free" intracellular MTX in suppressing actual DHFR activity, and the relationship between DHFR activity and UdR incorporation into DNA, remain undefined. Moreover, the role of DHFR-bound MTX in the absence of "free" drug is unclear. These studies have been undertaken to examine further the role of both "free" and bound MTX on the inhibition of DNA synthesis and on the

* Present address: School of Medicine University of Zambia, P.O. Box RW 110, Lusaka, Zambia.

activity of DHFR in L1210 ascites tumour *in vitro*.

### MATERIALS AND METHODS

*Chemicals.*—[3′, 5′, $^3$H]MTX was obtained from Amersham/Searle Corp., Arlington Heights, Ill. Unlabelled MTX was obtained from the Drug Development Branch of the National Cancer Institute. Both drugs were purified by linear-gradient elution with ammonium bicarbonate buffer on a DEAE-cellulose column as previously described (Goldman, Lichtenstein and Oliverio, 1968). Tritiated deoxyuridine ([6-$^3$H] UdR, sp. act., 21 Ci/mmol) and D-[4,5-$^3$H]leucine (sp. act., 1 Ci/mmol) were also obtained from Amersham/Searle Corp., Arlington Heights, Ill. Cycloheximide was obtained from the Sigma Chemical Co., St. Louis, Mo.

*Cells and media.*—L1210 leukaemia cells were grown i.p. in $CDF_1$ female mice. Animals were killed on Day 6 after inoculation of $10^5$ cells, and the ascitic tumour cells harvested by lavaging the peritoneal cavity with 4°C bicarbonate-buffered 0·85% NaCl (pH 7·4). The cells were freed of red-cell contamination by 30 sec hypotonic lysis and collected by centrifugation at 750 *g* for 5 min at 4°C. The cell pellet was resuspended in 4°C Eagle's minimal essential medium (MEM) without serum or folic acid, and viability assessed by trypan-blue exclusion. All preparations had >95% viability by this technique. Cell viability was also assessed at each experimental point by trypan-blue exclusion and found to exceed 90% throughout the experimental period.

*Incubation techniques.*—The cell suspensions were adjusted to a cell count of 1–2 × $10^7$/ml and placed in glass incubation flasks suspended in a 37°C water bath and continuously agitated by a mechanical stirrer. Before the addition of MTX, a stream of warmed, humidified 95% $O_2$/5% $CO_2$ was passed over the incubation mixture and the mixture pre-incubated for 5 min. MTX was then added to give a bath concentration of 3 $\mu$M. Following an incubation of 20 min, a period sufficient to saturate high-affinity intracellular MTX binding sites (Goldman *et al.*, 1968) cells were centrifuged at 750 *g* at 4°C to terminate the incubation. The cell pellet was washed ×2 in 4°C MEM and resuspended in a large volume of 37°C MEM containing radio-labelled MTX at 0·01, 0·05, 0·10, 0·50, 1·0, 3·0 and 10 $\mu$M. The cells were then incubated for 40 min, long enough for MTX to reach a steady state, as has been previously shown (Goldman *et al.*, 1968) and confirmed by preliminary studies with this L1210 cell line. Additional uptake studies were carried out at an MTX concentration of 0·05 $\mu$M for a period of up to 60 min. After uptake at 0·05 $\mu$M, cells were washed ×2 in 4°C MEM and resuspended in a large volume of 37°C MEM alone. MTX was allowed to efflux for 30 min.

Cells were sampled at 5 min intervals during uptake and efflux of MTX by withdrawing aliquots of cell suspension (2–4 ml) and rapidly isolating cell pellets by centrifugation, with aspiration of the supernatant, an aliquot of which was saved for determination of extracellular MTX concentrations. Pellets were washed ×2 in a 4°C 0·85% NaCl solution to remove extracellular MTX, and the resulting pellet was drawn up into a Pasteur pipette and extruded on to a flexible polyethylene disc. The cell pellet was dried overnight at 70°C to constant weight, removed from the oven, immediately separated from the disc, and weighed on a Cahn RG auto-electrobalance (Cahn Instruments, Inc., Paramount, Calif.). Correction for weight increases during exposure to air at room temperature was done by serial weight determinations and interpolation to the time of removal from the oven. Dry pellet weights ranged from 0·5 to 3·0 mg. The pellet was then placed in the bottom of a scintillation vial and digested in 1N KOH at 70°C for 1 h. After cooling to room temperature, 18 ml of a methanol-toluene scintillation fluid (700 ml of toluene, 300 ml of methanol, 3 g of PPO, and 100 mg of POPOP) were added, and the vials counted in a Searle Analytic Mark III liquid scintillation counter (Searle Analytic, Chicago, Ill.). The $^3$H-counting efficiency was 26% as determined by an external standard, and the quench variation between samples was negligible.

*Deoxyuridine studies.*—The incorporation of UdR into cellular DNA was studied in a manner similar to that described under "Incubation Techniques", except that non-radioactive, purified MTX was used in these studies. After a 20 min uptake, efflux was permitted for 40 min at all the concentrations detailed under "Incubation Techniques", before the addition of 50 $\mu$l of [$^3$H]UdR to give a final UdR concentration of 0·10 $\mu$M. Aliquots of cell suspension (2 ml) were then sampled at 5 min intervals, and incubation terminated by the addition of 2 ml of 20% trichloroacetic acid (TCA) at 4°C. The cells were centrifuged into a pellet, the supernatant aspirated and the pellet washed ×2 in 5% TCA at 4°C. Cell pellets were aspirated into Pasteur pipettes, extruded onto polyethylene discs, and processed as described earlier. Results are expressed as mg dry weight of the TCA precipitate. UdR studies at an MTX concentration of 0·05 μM were carried out in a manner identical to that described under "Incubation Techniques". After uptake periods of 5, 10, 20, 40, and 60 min, cells were washed ×2 and resuspended in 37°C MTX-free medium and 50 μl of [$^3$H]UdR added, to give a bath concentration of 0·10 μM. Aliquots were then sampled at 5 min intervals as described above. Identical studies were carried out in the presence of 1 mM cycloheximide, a potent inhibitor of protein synthesis, to prevent *de novo* synthesis of new DHFR during the experimental period. Cycloheximide was in the incubation bath during uptake, efflux, and UdR-incorporation studies. The ability of cycloheximide to inhibit protein synthesis was confirmed, by studying the incorporation of [$^3$H]leucine into the TCA precipitate of the cell suspension as described above.

A comparison between the radioactivity recovered in the TCA precipitate and that incorporated into DNA, as determined by the perchlorate-extraction method (Goldman, 1974) revealed that >90% of the radioactivity was incorporated into cellular DNA, confirming previous reports of the reliability of the TCA technique (Goldman, 1974).

*Enzymatic studies.*—Cell pellets were obtained after efflux for 3 μM MTX studies, and after uptake for 0·05 μM MTX studies, to examine the specific activity of DHFR at pH 7. Each pellet containing $10^9$ cells, as measured on a Coulter Counter Model F (Coulter Electronics, Inc., Hialeah, Fla.) was suspended in 2·4 ml of 4°C Tris-HCl buffer (0·05M, pH 7·0 containing 0·2M KCl). This constituted a 1:4 dilution of the intracellular volume, and accordingly underestimated the effect of MTX on DHFR activity. Cells were lysed by alternate freezing and thawing ×3 and centrifuged (27,000 *g*, at 4°C for 30 min). Complete recovery of all enzyme activity was confirmed after the third freeze-thaw procedure (Bender and Makula, 1976). The supernatant was recovered for assay of DHFR activity by a spectrophometric method (Perkins *et al.*, 1967). The assay mixture contained in 1 ml: Tris-HCl buffer at pH 7·0, 100 μmol; KCl, 150 μmol; NADPH (Sigma Chemical Co., St. Louis, Mo.) 0·1 μmol; and 0·05–0·2 ml of the enzyme extract. A pH of 7·0 was chosen as approximating to the intracellular pH. The reaction was initiated by the addition of 0·05 μmol of dihydrofolate, prepared from folic acid as described by Blakley (1960) containing 10 μmol of 2-mercaptoethanol. Enzyme activity was determined by measuring the decrease in absorbance at 340 nm using a Gilford spectrophotometer Model 2000 at 37°C. Enzyme activity is defined as μmol of dihydrofolate reduced/h/ml of enzyme extract. Alternatively, specific enzyme activity was determined as μmol of dihydrofolate reduced/h/mg protein, as determined by the method of Lowry *et al.* (1951). Concurrent controls were run on each experimental day and results expressed as a percent of control.

The relationship between intracellular DHFR and tightly bound intracellular MTX was determined by titrating the DHFR with purified MTX as previously described (Bender and Makula, 1976). The cell volumes used in this analysis were calculated by determining the cell wet and dry weights. The [$^{14}$C]inulin (New England Nuclear Corp., Boston, Mass.) space was used to determine the extracellular volume, as described in Goldman *et al.* (1968).

### RESULTS

### *Uptake and efflux of [$^3$H]MTX by L1210 leukaemia*

The total intracellular MTX content under each experimental condition is summarized in Table I. A non-exchangeable or "tighly bound" MTX fraction of 4·27 ± 0·02 nmol/g dry cell weight was determined. Subtracting this value from the total intracellular MTX determined at each extracellular MTX concentration ($[MTX]_e$) an exchangeable intracellular MTX value can be determined (see Table I). Dividing this number by the ratio of intracellular water to cell dry weight of 4·38 ± 0·17 determined for this L1210 cell line, the intracellular MTX concentration ($[MTX]_i$) can be determined. The $[MTX]_i$ at each $[MTX]_e$ are summarized

TABLE I.—*Intracellular MTX* ([*MTX*]$_e$) *Concentration at Various Extracellular Concentrations* ([*MTX*]$_e$)

| [MTX]$_e$ (μM) | Total intracellular MTX (nmol/g dry cell wt) | Exchangeable intracellular MTX (nmol/g dry cell wt) | [MTX]$_i$ (μM) |
|---|---|---|---|
| 10 | 28·60 ± 0·75* | 24·33 | 5·55 |
| 3 | 15·23 ± 0·55* | 10·96 | 2·50 |
| 1 | 9·57 ± 0·30* | 5·30 | 1·21 |
| 0·50 | 7·30 ± 0·28* | 3·03 | 0·69 |
| 0·10 | 4·92 ± 0·06* | 0·65 | 0·15 |
| 0·05 | 4·52 ± 0·04* | 0·25 | 0·014 |
| 0·01 | 4·30 ± 0·03 | 0·03 | 0·0068 |
| 0 | 4·27 ± 0·02 | 0 | 0 |

Values are the means of at least 3 experiments performed on different days. The exchangeable fraction is determined by subtracting the non-exchangeable intracellular drug from the total determined at steady state. Dividing the exchangeable fraction by the ratio of intracellular water to dry wt (4·38 ± 0·17) gives [MTX]$_i$.

* Significantly greater ($P < 0{\cdot}05$) than the non-exchangeable drug level of 4·27 ± 0·02 nmol/g dry cell wt, as determined by paired $t$ test.

in Table I. In each instance, high-affinity intracellular sites were saturated and "free" MTX was present in the intracellular volume, with the exception of zero [MTX]$_e$ studies.

Studies directed at subsaturation of high-affinity intracellular sites were carried out at a [MTX]$_e$ of 0·05 μM. Uptake was permitted for periods of up to 60 min, an interval insufficient to saturate these sites and over which uptake was linear (Fig. 1). Efflux studies were carried out to examine whether the intracellular MTX level declined after these cells were washed ×2 and resuspended in 37°C MTX-free MEM. No significant decline in intracellular MTX was observed over a 30 min period, confirming that intracellular drug levels were not declining during the period of UdR incorporation studies.

FIG. 1.—MTX uptake from 0·05 μM medium as a function of time. After various intervals, 30 min is illustrated, cells were washed ×2 and placed in MTX-free medium to allow efflux, and cells sampled at 10 min intervals for 30 min (dotted line, open circle). Each point represents the mean of at least 3 experiments.

*Incorporation of* [$^3$*H*]*UdR into the DNA of L*1210 *leukaemia cells*

Following MTX uptake and a 40 min efflux period, during which a steady state was reached, cell suspensions were pulsed with [$^3$H]UdR and incorporation permitted for a 30 min interval, over which incorporation was linear with time. Further, high [MTX]$_e$ did not alter the rate of UdR entry into cells, as has been previously demonstrated in Ehrlich ascites cells (Goldman, 1974). The UdR incorporation recorded under the experimental conditions is summarized in Table II.

UdR-incorporation studies were also carried out in the presence of cycloheximide to prevent *de novo* enzyme synthesis during the experimental period.

TABLE II.—*Deoxyuridine Incorporation under Each Experimental Condition*

| [MTX]$_e$ (μM) | UdR incorporation (% of Control) |
|---|---|
| 10 | 0·84 |
| 3 | 1·90 |
| 1 | 2·06 |
| 0·50 | 10·0 |
| 0·10 | 20·1 |
| 0·05 | 34·8 |
| 0·01 | 42·5 |
| 0 | 62 |
| 0·05 × 60′ | 90 |
| 0·05 × 40′ | 102 |
| 0·05 × 20′ | 132 |
| 0·05 × 10′ | 131 |
| 0·05 × 5′ | 120 |

Values are the means of at least 3 experiments on different days. Concurrent controls were always included with each experiment. The standard deviation of any mean value did not exceed 25%.

A 1 mM concentration was chosen, as preliminary studies suggested that [$^3$H]-leucine incorporation into the TCA precipitate of a cell suspension was inhibited by 93% over a 60 min period of exposure, comparable to the 20 min uptake and 40 min efflux periods used experimentally before the addition of UdR. Cycloheximide decreased the absolute UdR-incorporation rates under control and experimental conditions below the rates in MTX-containing cells not exposed to cycloheximide. However, the relative effect of increasing total intracellular MTX on UdR incorporation, as seen in Table II, remained unaltered. The decrease observed may reflect secondary inhibition of DNA synthesis at high cycloheximide concentrations.

*Enzymatic studies*

The DHFR-activity determinations in cell homogenates after exposure to MTX, under conditions detailed under "Uptake and Efflux . . .", are outlined in Table III. All activities were determined at pH 7 in pellets of $10^9$ cells, having an intracellular volume of 0·60 ml, on at least 3 different days. No DHFR activity could be detected at an $[MTX]_i > 1\ \mu M$, using maximum scale expansion (0–0·10 OD units) on the Gilford spectrophotometer, 200 μl aliquots of cell extract, excess dihydrofolate, and observation periods of up to 10 min. At an $[MTX]_i$ of 0·69 μM or less, a low but increasing level of enzyme activity was detected with decreasing $[MTX]_i$. When the $[MTX]_i$ was 0 and all intracellular high-affinity sites were saturated, the specific activity was 6·09% of control. This relationship is depicted graphically in Fig. 2, where UdR incorporation and DHFR activity were plotted against total intracellular MTX. The $[MTX]_i$ corresponding to each point is given in Table I. Control activities varied from 0·82 to 1·07 μmol/h/mg protein on different experimental days. With subsaturating quantities of MTX associated with DHFR, enzyme activity increased to 78·7% of control when only 16·6% of the enzyme

TABLE III.—*Enzyme Activities at Various Cellular MTX Levels*

| Total intracellular MTX (nmol/g dry cell wt) | DHFR sp. act. (μmol/h/mg protein) | DHFR activity (% of control) |
|---|---|---|
| 28·6 ± 0·75 | 0 ± 0 | 0 ± 0 |
| 15·2 ± 0·55 | 0 ± 0 | 0 ± 0 |
| 9·57 ± 0·30 | 0 ± 0 | 0 ± 0 |
| 7·30 ± 0·28 | 0·0202 ± 0·003 | 2·07 ± 0·21 |
| 4·92 ± 0·06 | 0·0233 ± 0·005 | 2·55 ± 0·17 |
| 4·52 ± 0·06 | 0·0405 ± 0·006 | 4·43 ± 0·14 |
| 4·30 ± 0·03 | 0·0454 ± 0·007 | 4·97 ± 0·06 |
| 4·27 ± 0·02 | 0·055 ± 0·011 | 6·09 ± 1·49 |
| 3·38 ± 0·11 | 0·12 ± 0·01 | 12·2 ± 0·62 |
| 2·57 ± 0·05 | 0·21 ± 0·01 | 21·5 ± 1·5 |
| 1·28 ± 0·04 | 0·51 ± 0·14 | 51·1 ± 9·0 |
| 0·71 ± 0·02 | 0·78 ± 0·11 | 78·7 ± 2·7 |
| 0·37 ± 0·02 | 0·76 ± 0·11 | 76·6 ± 2·5 |

Enzyme activities are the means ± s.d. of duplicate determinations of enzyme activity in cell pellets of $10^9$ cells prepared on at least 3 different experimental days. Concurrent controls were run on each experimental day. Total $[MTX]_i$ represents the mean ± s.e. of at least 3 experiments on different days.

sites (assuming a 1:1 MTX to DHFR association) were saturated. UdR incorporation was enhanced to 130% or more of control at these same points, (Figs. 2 and 3). The recovery of DNA synthesis is seen to occur concurrently with, but at a slower rate than, the recovery of DHFR activity. Maximal suppression of both DHFR activity and UdR incorporation appears to require "free" intracellular MTX. Conversely, both DHFR activity and UdR incorporation rapidly recovered and approached or exceeded control values, as fewer high-affinity sites were MTX-associated. When DHFR activity was undetectable, minimal UdR incorporation was still found (Fig. 2). However, when the DHFR activity recovered to 5% of control, UdR incorporation recovered to 50% of control, reaching 100% when DHFR activity was 20% of control (Fig. 3).

The relationship between high-affinity intracellular MTX and DHFR was examined by determing the $I_{50}$ values for MTX, and converting this amount of drug to μmol of DHFR/g dry cell wt, using modifications of a previously described method

FIG. 2.—UdR incorporation into DNA and DHFR activity at various total intracellular MTX contents. Each point represents the mean value of at least 3 experiments performed on different days. All points to the right of the vertical arrow represent total intracellular MTX in excess of high-affinity binding sites; those to the left of the arrow represent the converse.

FIG. 3.—The relationship between DHFR activity and UdR incorporation. The points are derived from data in Fig. 2.

(Bender and Makula, 1976). The $I_{50}$ to $I_{100}$ conversion required no modification, but our cell wet wt/dry wt ratio of 5·23 ± 0·49 required alteration of the cell number to dry wt conversion from that previously published (Bender and Makula, 1976). This method generated a mean DHFR content of 3·87 ± 0·41 μmol/g dry cell wt compared to a non-exchangeable MTX content of 4·27 ± 0·02 μmol/g dry cell wt. These values do not differ significantly by paired *t* test, confirming that the non-exchangeable MTX level corresponds to the cellular DHFR content.

### DISCUSSION

Methotrexate traverses the cell membranes of L1210 cells by an active transport mechanism (Goldman *et al.*, 1968) and accumulates in the intra-cellular space in at least 2 states. A constant amount appears bound to DHFR, and a variable quantity ("free" MTX) accumulates in excess of the bound amount. The role of both bound and "free" MTX has been examined herein. It has been shown *in vitro* that "free" drug is required maximally to suppress UdR incorporation into DNA and DHFR activity required for the reduction of dihydrofolate to tetrahydrofolate, a cofactor essential for 1-carbon metabolism. This observation is in agreement with the *in vitro* work of Goldman (1974), White and Goldman (1976), Roberts and Wodinsky (1968), Sirotnak and Donsbach (1974), and Borsa and Whitmore (1969). These investigators have reported maximal inhibitory $[MTX]_e$ of 3, 30, 0·9 and 1·0 $\mu$M, respectively, in several mammalian tumour cell lines. Sirotnak and Donsbach (1974) have expanded this view, by observing that less intracellular MTX was required *in vivo* to achieve the same effect, and observed that about 120 min of MTX exposure was required to achieve maximal suppression of UdR incorporation. However, inspection of their data reveals that 90% or more inhibition was already found by 60 min of exposure, the experimental period used in our studies. They conclude that the role of exchangeable intracellular drug may be to titrate MTX which slowly dissociates from DHFR binding.

The direct measurement of DHFR activity may circumvent the potential artifact introduced by the delayed effect of MTX on UdR incorporation. These studies require cell-free preparations, dihydrofolate concentrations in excess of those achieved physiologically, and a dilution of the intracellular volume. Accordingly, they are likely to underestimate the effect of a given $[MTX]_i$ on enzyme activity. Moreover, as the MTX concentration is static in these studies, and not subject to half-life decay and distribution pharmacodynamics as in an intact organism, this system may be a poor model for study of the *in vivo* interaction between MTX and DHFR in the intact cell. However, attempting to take these problems into account, our data reveal that UdR incorporation declines from 61% of control at the "equivalence point" where all high-affinity sites are saturated to 0·89% of control at a $[MTX]_e$ of 10 $\mu$M. However, DHFR activity drops to a less but statistically significant degree, from 6·09% of control at the "equivalence point" to zero at an $[MTX]_e$ or 1 $\mu$M or greater. The significance of this 6% change in DHFR activity remains speculative, but may be fundamental to the role of exchangeable intracellular MTX. If 6% of the enzyme activity is sufficient to support cellular functions, "free" MTX may be needed for maximal cytotoxicity. Conversely, if the 6% activity is insufficient for cell maintenance, only saturation of all high-affinity intracellular binding sites is needed for a maximal MTX effect. As 5% of the DHFR activity is sufficient to permit UdR incorporation to continue at 50% of control levels (Fig. 3) there is reason to suspect that "free" MTX is necessary. If, indeed, the MTX-DHFR association is slowly reversible, kinetic considerations would dictate that maximal inhibition of enzyme activity requires "free" drug in the intracellular fluid to keep all the enzyme sites saturated. Thus, although a $K_i$ of the order of $10^{-10}$ has been reported for MTX-DHFR in cell-free preparations (Bertino *et al.*, 1964; Werkheiser, 1961) for reductases from several sources, inability to determine this value in the intact cell poses a problem. A significant dissociation rate for MTX would explain the necessity for exchangeable intracellular drug. This view is consistent with the sigmoid relationship between total intracellular MTX and DHFR activity or UdR incorporation rates (Fig. 2). Such a curve is most compatible with a slowly reversible enzyme-inhibitor interaction in which some, but not all, inhibitor is enzyme bound (Straus and Goldstein, 1943). Whereas study of the DHFR-MTX interaction in cell-free systems at pH 5·9 suggests "stoichiometric" inhibition (Bertino *et al.*, 1964) the actual interaction occurring in the intact cell at physiological pH is more likely to be slowly reversible. An alternate explanation would require several forms of DHFR with different affinities for MTX. Although studies by Hangg and Littlefield (1974) suggest the existence of several species of DHFR in MTX-resistant hamster cells, their affinities for MTX have not been examined, and must remain the subject of speculation.

The likelihood of slowly reversible binding of MTX to DHFR is consistent with the observed efficacy of "high dose" MTX in certain human neoplasms. The low MTX permeability of those human tumours studied (Dedrick *et al.*, 1975) and inability of these cells to concentrate MTX intracellularly (Bender, 1975) makes them less likely to achieve "free" intracellular MTX at conventional doses. Thus, larger doses are needed to achieve higher $[MTX]_e$, to favour the accumulation of "free" intracellular drug. How high an $[MTX]_e$ must be achieved, and for how long it must be maintained, is likely to vary among tumours. Additional studies in intact cells and tumours will, we hope, provide these answers.

The authors would like to acknowledge the excellent technical assistance of Jane M. Pitts and James C. Drake.

### REFERENCES

BENDER, R. A. (1975) Membrane Transport of Methotrexate (NSC-740) in Human Neoplastic Cells. *Cancer Chemother. Rep.*, **6**, 73.

BENDER, R. A. & MAKULA, D. R. (1976) Relationship between Intracellular Dihydrofolate Reductase and Tightly Bound Intracellular Methotrexate in Human Neoplastic Cells. *Biochem. Pharmacol.*, **25**, 975.

BERTINO, J. R. (1963) The Mechanism of Action of the Folate Antagonists in Man. *Cancer Res.*, **23**, 1286.

BERTINO, J. R., BOOTH, B. A., BIEBER, A. L., CASHMORE, A. & SARTORELLI, A. C. (1964) Studies on the Inhibition of Dihydrofolate Reductase by the Folate Antagonists. *J. biol. Chem.*, **239**, 479.

BLAKLEY, R. L. (1960) Crystalline Dihydropteroylglutamic Acid. *Nature, Lond.*, **188**, 231.

BORSA, J. & WHITMORE, G. F. (1969) Studies Relating to the Mode of Action of Methotrexate. II. Studies on Sites of Action in L-Cells *In vitro*. *Mol. Pharmacol.*, **5**, 303.

DEDRICK, R. L., ZAHARKO, D. S., BENDER, R. A., BLEYER, W. A. & LUTZ, R. J. (1975) Pharmacokinetic Considerations on Resistance to Anticancer Drugs. *Cancer Chemother. Rep.*, **59**, 795.

GOLDMAN, I. D. (1974) The Mechanism of Action of Methotrexate. I. Interaction with a Low-Affinity Intracellular Site Required for Maximum Inhibition of Deoxyribonucleic Acid Synthesis in L-Cell Mouse Fibroblasts. *Mol. Pharmacol.*, **10**, 257.

GOLDMAN, I. D. & FYFE, M. J. (1974) The Mechanism of Action of Methotrexate. I. Augmentation by Vincristine of Inhibition of Deoxyribonucleic Acid Synthesis by Methotrexate in Ehrlich Ascites Tumor Cells. *Mol. Pharmacol.*, **10**, 275.

GOLDMAN, I. D., LICHTENSTEIN, N. S. & OLIVERIO, V. T. (1968) Carrier-mediated Transport of the Folic Acid Analogue, Methotrexate, in the L1210 Leukemia Cell. *J. biol. Chem.*, **243**, 5007.

HANGG, U. J. & LITTLEFIELD, J. W. (1974) Isolation and Characterization of the Multiple Forms of Dihydrofolate Reductase from Methotrexate-resistant Hamster Cells. *J. biol. Chem.*, **249**, 1390.

JAFFE, N., FREI, E., TRAGGIS, D. & BISHOP, Y. (1974) Adjuvant Methotrexate and Citrovorum-factor Treatment of Osteogenic Sarcoma. *New Engl. J. Med.*, **291**, 994.

LOWRY, O. H., ROSENBROUGH, N. J., FARR, A. L. & RANDALL, R. J. (1951) Protein Measurement with Folin Phenol Reagent. *J. biol. Chem.*, **193**, 265.

MARGOLIS, S., PHILLIPS, F. S. & STERNBERG, S. S. (1971) The Cytotoxicity of Methotrexate in Mouse Small Intestine in Relation to the Inhibition of Folic Acid Reductase and of DNA Synthesis. *Cancer Res.*, **31**, 2037.

MITCHELL, M. S., WAWRO, N. W., DECONTI, R. C., KAPLAN, S. R., PAPAC, R. & BERTINO, J. R. (1968) Effectiveness of High-dose Infusions of Methotrexate Followed by Leucovorin in Carcinoma of the Head and Neck. *Cancer Res.*, **28**, 1088.

PERKINS, J. P., HILLCOAT, B. L. & BERTINO, J. R. (1967) Dihydrofolate Reductase from a Resistant Subline of the L1210 Lymphoma. Purification and Properties. *J. biol. Chem.*, **242**, 4771.

ROBERTS, D. & WODINSKY, I. (1968) On the Poor Correlation Between the Inhibition by Methotrexate of Dihydrofolate Reductase and of Deoxynucleoside Incorporation into DNA. *Cancer Res.*, **28**, 1955.

SIROTNAK, F. M. & DONSBACH, R. C. (1974) The Intracellular Concentration Dependence of Antifolate Inhibition of DNA Synthesis in L1210 Leukemia Cells. *Cancer Res.*, **34**, 3332.

STRAUS, O. H. & GOLDSTEIN, A. (1943) Zone Behavior of Enzymes. Illustrated by the Effect of Dissociation Constant and Dilution on the System Cholinesterase-Physostigmine. *J. gen. Physiol.*, **26**, 559.

WERKHEISER, W. C. (1961) Specific Binding of 4-Amino Folic Acid Analogues by Folic Acid Reductase. *J. biol. Chem.*, **236**, 888.

WHITE, J. C. & GOLDMAN, I. D. (1976) Mechanism of Action of Methotrexate. IV. Free Intracellular Methotrexate Required to Suppress Dihydrofolate Reduction to Tetrahydrofolate by Ehrlich Ascites Tumor Cells *In vitro*. *Mol. Pharmacol.*, **12**, 711.